# Constructive eHealth evaluation: lessons from evaluation of EHR development in 4 Danish hospitals

**DOI:** 10.1186/s12911-017-0444-2

**Published:** 2017-04-20

**Authors:** Anna Marie Balling Høstgaard, Pernille Bertelsen, Christian Nøhr

**Affiliations:** 1 Department of Health Science and Technology, Aalborg University, Niels Jernesvej 14, 9220, Aalborg Øst, Aalborg, Denmark; 2 Department of Planning, Danish Centre for Health Informatics, Aalborg University, Vestre Havnepromenade 5, Aalborg, Denmark

**Keywords:** User involvement, End-user participation, Formative evaluation, eHealth development, Evaluation method, Constructive eHealth evaluation, Participation, End-user, Health information technology, Organizational factors

## Abstract

**Background:**

Information and communication sources in the healthcare sector are replaced with new eHealth technologies. This has led to problems arising from the lack of awareness of the importance of end-user involvement in eHealth development and of the difficulties caused by using traditional summative evaluation methods. The Constructive eHealth evaluation method (CeHEM) provides a solution to these problems by offering an evaluation framework for supporting and facilitating end-user involvement during all phases of eHealth development. The aim of this paper is to support this process by sharing experiences of the eHealth evaluation method used in the introduction of electronic health records (EHR) in the North Denmark Region of Denmark. It is the first time the fully developed method and the experiences on using the CeHEM in all five phases of a full lifecycle framework is presented.

**Methods:**

A case study evaluation of the EHR development process in the North Denmark Region was conducted from 2004 to 2010. The population consisted of clinicians, IT professionals, administrators, and vendors. The study involved 4 hospitals in the region. Data were collected using questionnaires, observations, interviews, and insight gathered from relevant documents.

**Results:**

The evaluation showed a need for a) Early involvement of clinicians, b) The best possible representation of clinicians, and c) Workload reduction for those involved. The consequences of not providing this were a lack of ownership of decisions and negative attitudes towards the clinical benefits related to these decisions.

Further, the result disclosed that by following the above recommendations, and by providing feedback to the 4 actor groups, the physicians’ involvement was improved. As a result they took ownership of decisions and gained a positive attitude to the clinical benefits.

**Conclusions:**

The CeHEM has proven successful in formative evaluation of EHR development and can point at important issues that need to be taken care of by management. The method provides a framework that takes care of feedback and learning during eHealth development. It can thus support successful eHealth development in a broader context while building on a well-known success factor: end-user involvement in eHealth development.

## Background

During the past few decades, centralised eHealth technologies have replaced previous information and communication sources throughout the healthcare sector at an ever-increasing rate in large parts of the world. The objective is to use information and communication technology (ICT) to improve patient health and safety and the quality of treatment. However, besides its benefits, this development has also resulted in challenges to the organization and to evaluation-methodologies.

Although challenges related to e.g., availability, structure, and overview of patient-related information may have been solved by the introduction of eHealth technologies, other problems of an organizational nature have been introduced, as the focus in eHealth development is often directed towards the technological aspects, while organizational aspects are underrated [1–7]. End-user involvement in design and implementation is an example of an important organizational issue that requires special attention for a successful outcome [2, 3, 6, 8–12]. Experience has shown such involvement to be a precondition for achieving the positive clinical benefits of new eHealth technologies [1, 10, 13], as well as joint ownership [11, 12, 14–16] - both well-known success factors in eHealth development. The importance of end-user involvement in technological development processes in general has been recognized since the 1950s, when members of the London Tavistock Institute formulated the “sociotechnical approach”, according to which both users and the technical aspects of work processes must be accommodated to achieve joint optimization [17, 18]. During the following decades this sociotechnical approach was further developed [18, 19], and Enid Mumford established the “ETHICS” method that integrated the approach into IT system development [9, 20]. According to Mumford, the overall objective is to ensure that equal weight is given to technical and human factors in the design of technology, and that future users are involved in the design process [9, 21]. However, the concept ‘end-user involvement’ spans a broad scale, from end-users acting as consultants during the late phases of the technological development lifecycle to end-users exerting influence on the decisions made during *all* phases in the lifecycle, from innovation to operation. Experience shows that, to achieve the success factors mentioned above, the involvement must be at the first, more comprehensive, end of the scale: end-users must be involved at the earliest possible stage as their ability to influence decisions already taken gradually decreases as the development process progresses [15, 22–25].

Although organizational issues in eHealth development have been widely discussed in recent decades, eHealth implementations continue to face problems caused by a lack of awareness of their importance [13, 26–28]. A recent example is given by the evaluation of the implementation of a new nationwide EHR system in England, which has displayed limited or no benefits, mainly because of the absence of user involvement in the decisions made during the development process [27]. This calls for new methods in the application of end-user involvement in the practice of eHealth development.

The evaluation-methodological challenges concerns the continued preference for summative methods which are facing fundamental difficulties as e.g., that the end goals must be fixed, the outcomes and the indicators be stable, and no intervening variables can be present [29–32]. However, eHealth development rarely fulfils these criteria, because a) eHealth development is a lengthy process as the technology often changes during the process, jeopardizing the evaluation methodologically; b) Traditional summative evaluation cannot be carried out until the technology is in operation - that is, when it is almost fully developed, making major changes difficult or expensive to implement should the evaluation show a need for this; and c) Traditional summative evaluation methods fail to accommodate the complexity of the healthcare sector with respect to differences in work practices across wards, clinical specialties, professional groups, etc. [22, 33, 34]. This calls for new methods for evaluating eHealth.

The new Constructive eHealth evaluation method (CeHEM) aims specifically to be used in evaluating eHealth in context, and offers a solution to the challenges outlined above. The method is inspired by the Constructive Technology Assessment (CTA) methods, which are formative evaluation methods to assess technology in general. The aim is to provide feedback on the strengths and weaknesses including the views of different actor groups, during the entire technological development lifecycle [35, 36]. The CTA concept, introduced by Leyten and Smits in 1987, has a specific focus on creating strong links between the technological development process and the user environment into which the technology is to be applied by establishing networks between relevant actor groups [37, 38], and shift the focus away from the various outcomes of fully developed technologies to also include relevant actor groups and the process itself [22, 33, 34]. The methods are typically inspired by interdisciplinary research perspectives from cognitive psychology, computer science, systems engineering, etc. [39]. During the 1990s, researchers in the Netherlands and Scandinavia in particular introduced CTA methods into the healthcare sector. The need for technology assessment increased significantly during these years because of the strong growth of technology in the sector. Thus, a number of experiences of using CTA methods within the healthcare sector were described during this decade [22, 40–48].

**Fig. 1:**
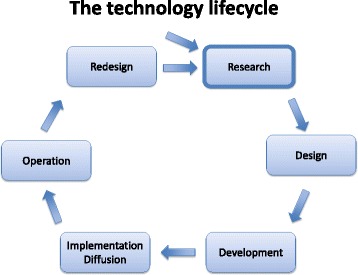
Phases in the eHealth development lifecycle (Figure inspired by [51]).Figure shows the eHealth development lifecycle as an on-going process, as it is necessary to adapt to changes in the needs and requirements over time, resulting in continuous redesigns of any technological systems until it might be phased out

Apart from these early experiences, CeHEM is grounded on the “sociotechnical carrier of technology theory”, which combines Müller et al.’s “sociotechnical theory” [22] and the “social carrier of technology theory” [49]. According to the combined theory, any change taking place during the technological development lifecycle can be traced to changes within the different actor groups or in their respective interests in the technology or capability to carry these interests through. Additionally, technology is viewed as having a mutual and lasting impact on society, which means that changes in technology will eventually result in changes in society, and vice versa [50, 51]. This view of technology as a social system is in line with Soft Systems Methodology (SSM) thinking as introduced by Checkland [52–54]. The CeHEM has been further developed over the past two decades, based on experiences from a number of evaluation studies within the Danish healthcare sector by researchers at the Danish Centre for Health Informatics (DaCHI) at Aalborg University (AAU) [55–59]. We employed a five-phased method based on the description by Müller et al. of the phases of eHealth development [51] combined with a lifecycle-inspired approach introduced in system engineering in the 1960s [60] (Fig. 1). The eHealth development lifecycle should be perceived as a nonlinear, iterative process without distinct phases [22, 51]. The method is characterized by its focus on supporting *end-user* involvement during *all* phases, the end-users being *direct* users of the eHealth system as defined by Barber, Cornford, et.al [61], i.e., clinicians in the healthcare sector.

To give the end-users as much influence as possible over the decisions taken, they should be involved in the CeHEM during the first eHealth development lifecycle [15, 22–25]. However, the method can also be used in lifecycle analysis to follow, if the evaluation involves the redesign of an eHealth system. This will of course considerably reduce end-user influence on decision-making.

### Introduction to the constructive ehealth evaluation method

The CeHEM offers a full-lifecycle evaluation framework to support and facilitate end-user involvement during the eHealth lifecycle. It includes methods to guide the process during all phases, and a modified summative evaluation. It draws attention to evaluation as an independent activity in parallel with other project activities.

As earlier publications have described the method in detail [62, 63], a brief description is given below.

### Preanalysis

The evaluation activities are prefaced by contextual evaluation questions: “what, why, when, who, and how”. If available data do not allow these questions to be answered, the evaluation should not start [64, 65].

### Phase 1: Research and planning phase

- ***1a) Identification of relevant actor groups:*** Obvious actor groups in eHealth development include: clinicians, management, vendors, and IT professionals. However, to identify all relevant groups, partner analysis is recommended [66, 67].
- ***1b) Selection of representatives of the identified groups:*** In order to support the achievements of ownership of the decisions taken during the eHealth development lifecycle, representatives of the various actor groups should be identified and chosen by peers [8, 16]. At the regional and hospital level, it is recommended that the professional associations make this selection. At ward level, the participatory method can be recommended [68].
- ***1c) Technology-carrier analysis:*** Identifying possible conflicts of interests and carrying out power analyses of the interactions between actor groups can be an important part of Phase 1. To secure influence, six conditions need to be fulfilled in relation to the technology in question: interest in the technology, the power to carry through these interests, an organization to support them, and information on, access to, and knowledge of the technology [50].

### Phase 2: Design phase

- ***2a. Identifying needs:*** Needs analysis of basic functionalities should be carried out to identify the requirements that the new eHealth technology must meet.
- ***2b. Requirement specifications:*** In 2005 the European Union’s Competitive Dialogue Process, was introduced as a new way of handling procurement. This method aims at highly complex areas where the provider (the eHealth project management) has difficulties in defining legal, technical, and/or financial matters related to the project. To ensure better-informed decisions, the Competitive Dialogue Process allows end-users to pose questions to the vendors on technical and other matters and hence supports end-user involvement in decision-making.

### Phase 3: Development phase

The needs and requirements from Phase 2 are here converted into a first version of the new eHealth system, a pre-prototype. This process is made from collaboration and feedback between involved stakeholders. The User Innovation Management method, which is inspired by user-centred and participatory design methods [69–71] is recommended to facilitate the outlines of the possible technical configurations of the new eHealth system, as this method supports end-user involvement in the design of a new technology [72]. The pre-prototype is tested in usability laboratories, simulation labs or in clinical set-ups to assess, for example, user-friendliness in work practice. Jacob Nielsen’s ten heuristics for usability studies may be used [73]. The outcome of Phase 3 is a prototype.

### Phase 4: Implementation and diffusion phase

Here the prototype is implemented for test in different *clinical* contexts. Working groups are formed at ward level to customize the prototype to specific clinical needs. The methods recommended for Phase 1b also apply here. During this phase, the system is tested when used in daily clinical practice. The test in clinical practise continues until the feedback between the ward working group has shown that ward-level customization is fulfilled and that end-users have accepted the result.

### Phase 5: Modified summative evaluation

The modified summative evaluation includes the measurement of a) The before–after technical and/or economic quantitative changes and/or b) Qualitative and quantitative assessments of organizational changes - e.g., end-user satisfaction. In contrast to traditional summative evaluation methods, not all of the outcome measures and indicators in the CeHEM are unchangeable from Phase 1. The objective of the evaluation will guide which measurements are included, which are unchanged and which are reconsidered during Phase 1–4. In large eHealth development processes, it is difficult to predict which outcomes and indicators are relevant – especially for those of an organizational nature - until an actual prototype has been implemented, customized, and tested in a specific context because of, for example, the length of the process and the organizational complexity of healthcare. Thus, these outcome measures and indicators are not decided on until the prototype is customized, tested and retested, and accepted by all actors - i.e., in the last part of Phase 4 - with a special focus on the end-users. When this is reached, all outcome measures and indicators need to be fixed, and, for methodological reasons, the eHealth system should not be further adjusted until the modified summative evaluation is completed. While the technical and economic aspects of the summative evaluation should take place at national, regional, or hospital level (depending on the objective of the evaluation), the end-user assessment is best performed at ward level on account of differences between wards with respect to clinical specialties, professional groups, and work practices.

The overall responsibility of the evaluator is to actively facilitate learning and feedback between the relevant actor groups during all phases in the eHealth development lifecycle, in particular focusing on end-users. The evaluator’s role is thus to ensure that the end-users are meaningfully involved in decision-making in all phases of the lifecycle.

Conflicts of interest may occur during the process if the evaluator or the evaluation team is identical with the project manager or project management [74] (Table 1).

**Table 1:** The Constructive eHealth Evaluation Method (CeHEM): phases, objective, activity, and methods

Phase	Objective	Activity	Methods
Pre-analysis	Justify a constructive evaluation need	Identify: Why… When… Who… How… Health care Management meetings based on results from summative evaluation of existing eHealth system performance	Meetings Data assessment Document analysis Satisfaction survey
Research and planning	Set-up a competent project organisation to provide solid health care and ICT professional insight to management and CIO	a) Identification of relevant actor groups b) Selection of representatives of the identified groups c) Technology carrier analysis d) Identify indicators for final summative evaluation	Interviews Observation of work practices When users identify users Document analysis Survey
Design	Identify appropriate system developer based on the identified requirement specifications	Identifying user needs Draft requirement specifications Select vendors	User centred and participatory design methods e.g., User Innovation Management method (UIM)
Development	Design of proto type adjust and re-design	Information meetings with department management from different clinical context Select appropriate clinical context/wards for pilot test Pilot test of Pre-prototype Feed-back structure for errors and changes re-design	Meetings with management from different clinical context Presentation of prototype Selected Interviews for feedback Observation of test
Implementation and diffusion	Test of proto-type in clinical setting	Form working group at department and/or ward level a) Identification of local actor groups relevant for the clinical settings b) Selection of representatives of the identified groups Customise prototype Test prototype functionalities Test usability	Clinical simulation Think aloud Cognitive walkthrough Observation of work practices
Modified summative evaluation	Before and after technical, organizational and economic changes	End user assessment Measure based on indicators	Qualitative investigation Quantitative survey

### Other formative evaluation methods

Existing formative evaluation methods have a different focus, and are covering only parts of the eHealth lifecycle or differ from the CeHEM in other ways.

In the 1990s, Bødker et al. introduced the MUST Method for Participatory Design, aimed at supporting end-user involvement in information technology development. However, it focused solely on supporting end-users during the design phase. Besides, the method focuses on ICT development in general, as it is intended to support IT professionals responsible for the design phase [14, 75].

Kaufman et al. have suggested a full-lifecycle evaluation framework for eHealth development in which a five-stage development model is presented. The framework, which has a user-centred approach to design, aims to provide a heuristic for matching the stages of system design and the levels of evaluation. In contrast to the CeHEM, the main focus is not on supporting end-user involvement, but rather on structuring research in health informatics by providing a rigorous evaluation at each stage in the lifecycle [76].

Full-lifecycle frameworks targeting eHealth evaluation have also been proposed by Catwell and Sheikh, Clarke et al., and van Gemert-Pijnen. However, their methods differ from the CeHEM in several ways. Focusing on improving eHealth development or on implementation, they provide feedback between all groups involved, without any specific focus on end-users [5, 32, 77]. Besides, no tools are provided to guide the process through the different phases of eHealth development and, as has been emphasized by both Nykänen et al. [74] and Høstgaard [50], they do not support evaluation as an independent activity that takes place in parallel with other project activities.

Greenhalgh and Russell have offered a different approach based on ten alternative principles for eHealth evaluation [78]. Their principles are generally aligned with those of the CeHEM, but are presented in an abstract and generalized way, as they invite other researchers to help refine them.

Harrison et al. offer a new framework: the ISTA (Interactive Sociotechnical Analysis) framework and a typology specifying important relationships between new e-Health, workflows, clinicians, and organizations. The framework emphasizes the potential of these relations to produce unintended consequences. The objective is to make clinicians, managers, and designers aware of the risk of such unfolding consequences and to spot them through formative evaluation focused at the so-called “HIT in use” during implementation [79]. This framework differs from the CeHEM in several ways, as it is not a life-cycle evaluation framework in the proper sense but an interactive sociotechnical analysis.

Actor Network Theory has been used to evaluate complex IT-systems in healthcare service organisations [80]. They have proven useful in appreciating the complexity of reality and to understand social effects among different actors in a network. Their particular strength is of analytic nature, but provides little guidance for participatory activities.

Cresswell, Bates, and Sheikh advocate a lifecycle perspective of eHealth development - that is, formative evaluation methods - as a solution to the problems caused by traditional summative methods. They suggest ten important considerations for eHealth developments to succeed, focusing on involving end-users throughout the eHealth lifecycle to achieve co-ownership and commitment to the outcomes of the eHealth development process [2]. Their considerations are fully in line with the view on eHealth development on which the CeHEM is based Table 1.

The CeHEM offers a structured analytical framework and methods for meeting the need for new eHealth evaluation methods, as well as the need for end-user involvement in eHealth development and implementation. The importance of involving end-users has been increasingly widely recognized during recent decades [2, 4, 14, 16, 50, 81]. This is underpinned by the European Union’s newly published eHealth Task Force Report, “Redesigning Health in Europe in 2020”, in which a recommendation of end-user involvement features strongly [82]. Hence, the objective of this paper is to support successful eHealth innovation, development, and implementation by sharing the experiences gained from using the CeHEM during the evaluation of the EHR development process at regional level in the North Denmark Region in Denmark. This paper presents for the first time the fully developed method and the experiences on using the Constructive eHealth evaluation method during all five phases of the method.

## Methods

In Denmark, hospitals are public, financed by taxation and governed by the five regional authorities, each of which has their own EHR based on different development strategies and platforms. The Constructive eHealth evaluation method was used in a case-study evaluation during the development of a new EHR in the North Denmark Region. The authors were commissioned by the regional IT board to carry out Phases 1–4 of the CeHEM and the end-user assessment part of Phase 5. The technical and economic parts of Phase 5 were performed by the EHR project management and the regional IT department. The detailed results are confidential and available only to the regional IT board and the EHR project management.

### Design

The design was a case-study evaluation of the first EHR development lifecycle in the region using the CeHEM. The case object was all phases in the first EHR development lifecycle in the region, and was defined in time from 2004 to 2010 and in place as described below in the sub-section: “Setting” [83].

### Objective

The objective of the formative evaluation (Phases 1–4) was to support feedback between the identified actor groups - with a specific focus on the clinicians - to assist learning and to support end-user involvement. The objective of the modified summative evaluation (Phase 5) was to provide a survey of the clinicians’ assessment of the clinical benefits of the new EHR after implementation.

### Population

Identifying the relevant actor groups and recruiting participants is part of Phase 1 in the CeHEM, and is described in the Results section. In the present study, the term “clinicians” refers to physicians, nurses, and midwifes. However, there was a specific focus on the physicians, as several studies have demonstrated the importance of this end-user group to successful EHR development in that their acceptance of the new system is crucial to whether or not it is applied as intended in clinical work practice (Nøhr C, Høstgaard AM, Botin L, Kjær-Andersen S: Evaluering af GEPKA- projektet, Delrapport 2, Klinisk afprøvning, unpublished; [84, 85]). In Denmark, all physicians, including senior physicians, are expected to enter data into the EHR themselves as part of their work practice.

The actor groups included in the individual phases of the evaluation study were as follows:

- **Phases 1 and 2**: Clinicians (4 nurses, 2 physicians; after 18 months, 8 physicians), secretaries (1), IT professionals (8), administrators (the regional IT board (12 members of whom 2 were clinicians) and the EHR project management (5 members of whom 1 was a clinician)), and vendors (4). The clinicians, the secretary, and the IT professionals were all members of the so-called “EHR working group”, which was established at the start of Phase 1 with a responsibility for carrying out tasks from Phases 1 and 2.
- **Phase 3**: All ward-level clinicians and secretaries, IT professionals (3), administrators (the regional IT board, the EHR project management), and a vendor (1).
- **Phase 4**: All ward-level clinicians and secretaries, four IT professionals, administrators (the regional IT board and the EHR project management), and a vendor (1).
- **Phase 5** ***:*** All clinicians participating in Phase 4.

### Setting

- **Phases 1 and 2**: An office at Aalborg Hospital (meetings between the EHR working group and the vendors, one at a time)
- **Phase 3**: One ward at Aalborg Hospital
- **Phase 4**: Two wards at Aalborg Hospital, one ward at Frederikshavn Hospital, Frederikshavn, one ward at Himmerland Hospital, Hobro, and one ward at Himmerland Hospital, Farsø (either medical or surgical wards).
- **Phase 5:** All Phase 4 wards.

### Data collection for the formative evaluation, Phases 1–4

Data on the formative evaluation were collected using observation, interviews, and documents analysis. The overall perspective was on the interactions between the clinicians and the new EHR. However, the focus changed from phase to phase, as is elaborated below.

### Observations

During Phases 1 and 2, non-participant observations were carried out at all EHR working group meetings (19 in total). During Phase 3, non-participant observation was carried out at one ward, and during Phase 4 at four wards, supplemented by non-participant video observation. During Phases 1 and 2, the various interests and the balance of power between the different actor groups in the meetings were in focus, particularly with respect to the physicians. During Phases 3 and 4, the focus was on the interactions between the clinicians and the new EHR. This was studied during morning and afternoon shifts in the case of the physicians, at morning and afternoon clinical handovers in the case of the nurses, and during ward rounds for both physicians and nurses. For Phases 3 and 4, an observation guide was developed on the basis of preliminary visits to the respective wards to help the researchers maintain a shared perspective and focus. During these phases, observation notes were compared and discussed among the researchers immediately after completion of the observations. In case of disagreement, consensus was reached by discussion.

### Interviews

During Phases 1 and 2, we did semi-structured interviews with 11 physicians, two IT professionals, and two administrators. During Phases 3 and 4, we did two focus group interviews at each ward, with six to eight informants in each session. The interviews in Phases 1–2 and Phases 3–4 had the same focus as the observations. All interviews were informed by an interview guide that had been pilot-tested on individuals comparable to the informants and revised before use in the study. All interviews lasted 15–60 min and were recorded and fully transcribed. All transcriptions were validated by the informants. No requests for changes were made.

### Document access

During phase 1–5, the researchers had access to relevant documents including minutes of meetings in the EHR working group, project plans, and procurement documents from vendors.

### Data collection for the modified summative evaluation, Phase 5

#### Surveys

In the case of the North Denmark region, the objective of the modified summative evaluation was to provide a survey of the clinicians’ assessment of the clinical benefits of the new EHR after implementation. Thus, no actual before-after study was requested. Two surveys were conducted during Phase 5, one at the start and one at the end of Phase 4, in all five wards participating in Phase 4. The first survey aimed at measuring clinicians’ expectations of the clinical benefits of the new EHR prior to its implementation. The questionnaire was divided into: 1) Basic information on the informants; 2) Questions relating to the outcome measure: knowledge of the new EHR prior to implementation (the questions concerned indicators of this, with space provided for open answers); and 3) Questions relating to the outcome measure: the clinicians’ expectations of the clinical benefits of the new EHR (the questions concerned indicators of this, with space provided for open answers). The second survey measured clinicians’ assessment of the clinical benefits of the EHR after its implementation. The questionnaire for this survey was divided into: 1) Basic information; 2) Questions relating to the overall outcome measure - i.e., the clinical benefits of the new EHR, as measured by questions concerning the following indicators: the accessibility of clinical data, system performance, customization, and effectiveness. The formulation of indicators was guided by the objective of the survey, responses to the first survey, and experience from previous healthcare evaluation studies. Before use in the study, both questionnaires were pilot-tested on groups comparable to the respondents and revised.

### Data analysis for the formative evaluation, Phases 1–4

All data collected for the formative evaluation were analysed using a hermeneutic strategy [86]. All three authors collaborated on the analysis. In case of disagreement, consensus was reached by discussion. The ATLAS software program was used for structuring the analysis process [87].

### Data analysis for the modified summative evaluation, Phase 5

Frequency analyses of the physicians’ and the nurses’ expectations of the new EHR (first survey) and of their assessment of its clinical benefits (second survey) were performed at ward level using the statistical program SPSS. The open answers were analysed according to the strategy described for the formative evaluation.

## Results

The results of the evaluation in the North Denmark Region using the Constructive eHealth evaluation method are specified phase by phase. The evaluation of Phases 1 and 2 focused on the communications between the actor groups, specifically on the physicians. However, the evaluators (the authors) were not actively involved in the evaluation of these phases, in contravention of the ideas of the CeHEM. The results and the discussion provided for Phases 1 and 2 therefore focus on the actions actually taken during these phases, as compared to the recommendations in the CeHEM. Phases 3, 4 and 5 were done following the CeHEM.

### Preanalysis

The responses to the questions of “what, why, when, who, and how” are provided in the Methods section.

### Phase 1: Research and planning phase

- ***1a) Identification of relevant actor groups and 1b) selection of representatives of these groups:*** The CeHEM recommendations for Phases 1a and 1b were not followed in this case study. The EHR working group was established by the project management, who selected its members at the start of Phase 1 based on the staffs’ experience with participation in previous IT projects in the region. The group included clinicians (2 physicians and 4 nurses), secretaries (1), and IT professionals (8). Eighteen months later, the physicians’ professional organization at the hospital objected to all medical specialties, age levels, and locations only being represented by two colleagues. They feared that physician’s professional interests were not adequately represented. As a result, the group was supplemented with six physicians selected by their regional association. However, it was difficult for the newcomers. They had little chance to catch up with the knowledge acquired by the rest of the group during the previous 18 months - that is, to acquaint themselves with the extensive and very technical procurement material. This meant that they did not feel competent to make informed decisions. Thus, their decisions were primarily based on advice from the IT professionals. This caused the physicians to lack ownership [50]. As a consequence of a decision taken by the regional IT board workload reduction was not an option. Hence, the physicians were expected to work and as an extra task do the work associated with the EHR development process. This was possible only for the senior physicians at Aalborg Hospital - and only partly in their case - who could plan their daily work more flexibly than their younger colleagues and who did not have to spend time on transport to the meetings, which were held at Aalborg Hospital. As a consequence, seven out of the eight physicians in the working group were senior physicians from Aalborg Hospital. Time pressure caused the single physician who worked at a smaller regional hospital to leave the group after attending only one meeting. Younger physicians, who make up more than half of the physicians in the region, and physicians from the smaller regional hospitals, thus had no representation during Phases 1 and 2 [50].
- ***1c) Technology-carrier analysis*** The technology-carrier analysis was not performed as a part of Phase 1 - that is, before the need assessment for the new eHealth technology had been carried out, as prescribed by the CeHEM, but during Phase 2a (identifying needs). This will be dealt with in the Discussion section. Only a synthesis of the individual analysis of the six conditions required for true involvement in the development of the new EHR in the region will be provided in this paper, as a detailed analysis has previously been given [88]. The synthesis shows that the physicians did not become truly involved in the process, mainly because of their workload, which affected all six conditions required. Time pressures thus had the effect of reducing their role to that of informing the group of clinical needs. Hence they exerted little influence on most decisions made during Phases 1 and 2 of the EHR development lifecycle [50].

### Phase 2: Design phase

- ***2a) Identifying needs*** The evaluation revealed differences in the interest shown by the three main actor groups (clinicians, IT professionals, and administrators) in the basic functionalities of the new EHR. The main interest of the clinicians (and of the physicians in particular) was the clinical benefits and high user-friendliness. In contrast, IT professionals focused on the administrative functions, which were in line with the interest of the administrators, who focused on compliance with national requirements for clinical data handling. This means that, although the groups shared the same overall vision - that the new EHR improved patient health and safety and the quality of treatment - their identification of needs for the basic functionalities reflected their own interests. However, because of their underrepresentation during the first 18 months, the physicians were unable to specify their clinical interests. As a result, the clinical interests were formulated by a nurse administrator with clinical background and IT professionals working full-time on the EHR development project. The requirement specifications were subsequently formulated on this basis [50].
- ***2b) Requirement specifications*** The detailed requirement specifications for the new EHR were formulated through a Competitive Dialogue Process aimed at complex IT projects. This allowed all participants in the working group to ask questions of the vendors until they felt they were capable of making informed decisions. The Competitive Dialogue Process consisted of four phases:

1. The prequalification phase during which the EHR project management invited vendors to apply for participation in the process. Based on criteria formulated by the EHR project management, four vendors were invited to compete against each other in developing quotations for the new EHR.
2. The dialogue phase, involving the participants in the EHR working group and each of the four vendors, one at a time.
3. The quotation phase, during which provisional quotations for the new EHR were presented by each of the four vendors based on discussions in the dialogue phase. Based on these quotations, the dialogue continued until the working group had no more questions after which they formulated their final requirement specifications.
4. The decision phase, in which the four vendors tendered their final quotations.

The requirement specification phase was completed as the regional IT board selected the supplier on the basis of the working group’s recommendations and certain other criteria, e.g., economic. Having fulfilled its task, the working group was dissolved [50].

The support of end-user involvement enabled by the Competitive Dialogue Process ensures that it is in line with the CeHEM. The clinicians, in particular the physicians, could thus acquaint themselves with technological issues through dialogue with vendors. However, despite their very positive attitude to the Competitive Dialogue Process, the consequences of the physicians’ lack of real influence during Phases 1 and 2a were evident throughout. Hence, their choice of vendor relied heavily on advice from the group’s IT professionals and, as a consequence, the physicians felt little ownership of this decision [50].

### Phase 3: Development phase

During the development phase, a new working group was established at one ward. The representatives were chosen by and among management and staff. The group developed a pre-prototype in preparation for the prototype proper. Different functionalities (e.g., the user interface) were designed and developed using mock-ups and games (inspired by the User Innovation Management method [72]) in close collaboration between the actor groups; these were tested and retested on the basis of feedback and learning facilitated by the evaluators. The feedback took place through personal contacts, meetings, and a number of reports to the EHR project management and the IT board. This process continued for several months until all actors involved - the physicians in particular - had accepted the result: an actual prototype of the new EHR (Høstgaard AM: Evaluering af Præpilottest af “Klinisk Proces” på Infektionsmedicinsk afd. Aalborg Sygehus Syd, unpublished).

### Phase 4: Implementation and diffusion phase

During Phase 4, local working groups were established at five wards to customize and test the EHR prototype developed during Phase 3. Also during this process, the clinicians chose their own representatives, who all had clinical experience and interest in joining the working group. Also in this phase, the primary responsibility of the evaluators was to facilitate feedback between the different actor groups. The feedback resulted in important learning on a number of occasions: 1) In one ward, the evaluation revealed that an essential group of actors had not been included in the working group, due to an oversight by the project management. As a result, an indispensable clinical work document (the Partogram) had not been included in the EHR customized to this ward, which meant that this professional group could not carry out their daily clinical work when testing the prototype:

*“For us the Partogram is the main work document, where we enter our record notes – that is the Partogram, and it is not included (in the EHR)”* (midwife at focus group interview).

*“And this program (the Partogram) is the most important”* (midwife at focus group interview).

However, based on the feedback provided by the evaluators, the document was inserted into the EHR and the group included in the working group. 2) At another ward, a very long loading time for the EHR system at morning shifts meant that it had not been accepted. The evaluation revealed that a recent reorganization of work procedures had led to the omission of a key task formerly performed by the night shift: They rebooted all computers during their shift which made the computers perform faster and more efficiently during the morning shift.

*“…it (rebooting all computers) was an integrated part of the secretary’s work tasks at night shifts, when there were two….then some reorganization took place, and suddenly there is only one secretary at night, and she has plenty to do, because she has functions related to other wards as well as a consultant function for the entire hospital. Thus, it just fell out, and nobody took any notice of it, which means, that the computers are no longer rebooted”* (nurse at focus group interview).

When this was reported to the EHR project management, the procedure was reintroduced. 3) A pocket-size EHR system guide responding to most of the staff’s uncertainties about the new EHR was not being used. The evaluation showed that the users’ faith in the guide had suffered because a number of identified errors in the guide previously identified and reported by the staff involved had not been corrected.

*“… many times it (the pocket-size EHR system guide) says: choose something, and then it is in fact a sub-thing beneath the thing, it tells you to choose – do you understand? It is not comprehensive, and it skips several steps”* (nurse at focus group interview).

When the evaluators reported this problem to the EHR project management, a procedure for correcting mistakes in the guide supporting the system was established [89].

Phase 4 went on at all five wards until user feedback had convinced the management that the respective prototypes were ready for the summative evaluation in Phase 5.

### Phase 5: Modified summative evaluation

The modified summative evaluation took place at each of the five wards participating in Phase 4. Thus, when the EHR prototype had been customized, tested and retested, and finally accepted by the end-users at the five wards, no more changes of the outcome measures and indicators - and no further adjustment of the prototypes - took place until the modified summative evaluation was completed. The response rate in the first survey at the start of Phase 4 (which had the aim of identifying the clinicians’ expectations of the system) varied across the five wards; for physicians between 39 and 100%, for nurses between 41 and 53%. The results indicated three key issues across wards and professions: fast and easy access to clinical data, good overview of clinical data, and access to the EHR from multiple PCs. The second survey was carried out at the end of Phase 4 and aimed to establish the clinicians’ assessment of the clinical benefits of the new EHR; the physicians’ response rate varied between 35 and 77%, the nurses’ between 27 and 43%. Across all five wards, the results showed less positive attitudes among the physicians than among the nurses with respect to the following indicators: accessibility of clinical data, system performance, and effectiveness - with no correlation between the response rate and attitudes. At three of the five wards, the physicians were significantly less positive than the nurses; at two of these, the system was not used during ward rounds, only for access to historical data in acute admissions, while at the last, the physicians did not use the system at all because they felt it hampered their clinical work. At the remaining two of the five wards, the system was used as intended although the physicians were slightly less positive than the nurses. With respect to the last indicator, customization, the physicians and the nurses were equally positive - with a tendency for the physicians to be the most positive.

Besides the results mentioned above, the evaluation showed how a number of technical problems persisted, relating for example to log-on procedures, uptimes, and response times. Therefore, the evaluators (the authors) final recommendation to the management was to postpone further implementation and diffusion of the new EHR to other hospital wards until all these issues had been resolved - and hence, take no further steps until the system was able to meet the clinical needs. The management decided to wait for another year before implementing the system at all hospital wards in the region. During this year, several functionalities were adjusted. This may be assumed to have prevented a number of problems from occurring at later stages [89]. Although the implementation started before 2010 no final overall summative evaluation of the EHR development process in the North Denmark Region has yet - in 2016 - taken place.

## Discussion

### Discussion of results

The evaluation of Phases 1 and 2 was not a Constructive eHealth evaluation in the strict sense, as the evaluators were not actively involved and the technology-carrier analysis was performed as part of Phase 2a rather than as part of Phase 1c. However, the results of the evaluation strongly support the recommendation of the Constructive eHealth evaluation method. The case study not only points out when the involvement of the stakeholders are omitted but also specifically recall what the consequences can be. This is a natural result of an iterative development of a methodology. The results thus support the experiences gained from earlier empirical studies by DaCHI (Høstgaard AM: Evaluering af Præpilottest af “Klinisk Proces” på Infektionsmedicinsk afdeling Aalborg Sygehus Syd, unpublished [88–91]) and from the literature on the preconditions for achieving *real* involvement of the clinicians. The important prerequisites for achieving joint ownership of decisions taken during eHealth development thus appear to be: a) Early involvement of clinicians [15, 22–25], b) The best possible representation of all groups of clinicians [8, 9, 92], and c) Workload reduction [50, 93]. The evaluation revealed that the physicians in the EHR working group gained little ownership of the decisions taken during phase 1 and 2 because the above pre-requisites were not met. The use of the Competitive Dialogue Process in Phase 2b, which supported the physicians’ involvement, had little effect on the overall result of the evaluation. We argue that most of the problems caused by not involving enough physicians from different specialities could have been avoided if the recommendations of the CeHEM had been followed. This also appears to be relevant in particular to early participation, to the methods for identifying and selecting participants of the EHR working group and to the support given by the evaluators to the physicians during this process. Thus, a higher degree of ownership among the physicians of the decisions taken - a well-known success factor in eHealth development - could probably have been achieved - despite the absence of workload reduction.

The results of the evaluation also indicate a need to perform a technology-carrier analysis in Phase 1c - that is, before the needs for the basic functionalities in the new EHR are defined in Phase 2a. In the case of the North Denmark Region, it is very important to note that most of the physicians’ clinical needs were formulated by non-physicians, with a great risk that the group’s needs were not fully met, as experience shows that only the members of a professional group can formulate the specific needs of that group [8, 71, 94]. We argue that this risk could have been significantly diminished if the EHR project management had been alerted to the results of the technology-carrier analysis at an earlier stage, as such results have been shown to be good markers of end-users’ ability to show real involvement in decision-making in subsequent phases [50]. This argument is based on the observation that the regional IT board and the EHR project management were very responsive to the potential consequences of the physicians’ lack of ownership of the decisions taken. This was evident when, at the end of Phase 2, they were presented with the results of the evaluation of Phases 1 and 2, which revealed their poor involvement. This caused the leadership to focus strongly on supporting the clinicians’ involvement during the following phases by letting them chose their own representatives and having them all participate from the start. Although workload reduction was not an option, this did not affect the results of the evaluation to the same extent because the demands put on their time were less heavy than in the earlier phases.

During Phases 3 and 4, the evaluator-facilitated feedback between the actor groups gave emphasis to the physicians’ role. As a result, they were more heavily involved in the design of the prototype and its customization in different wards. Their ownership of the decisions taken during this phase was likewise much stronger [89]. The evaluators’ role during these phases was very important, as it most certainly prevented a number of problems from occurring later. Moreover, the experiences gained in the evaluation of Phase 4 showed the importance of using external evaluator/evaluators in order that responsibility for the evaluation is separated from other roles in the project and the hospitals to avoid conflicts of interest. Had the evaluation been an intrinsic part of the overall project activities, some of the problems identified would likely not have been discussed openly. This also stresses the importance of constructive exchange of feedback between actor groups, as this is a precondition for learning from the mistakes, rather than repeating them.

The less positive attitudes of physicians (in contrast to nurses) revealed by the summative evaluation applied to all five wards and were concerned with such basic functionalities of the EHR as accessibility to clinical data, system performance, and effectiveness. At three wards, the physicians’ attitudes to these indicators were negative. During Phases 1 and 2, it was decided which indicators to use. At that time, the physicians’ involvement in decision-making was very limited. Thus, even though no cause and effect relationship can be claimed, the summative evaluation show negative consequences of having the needs analysis and the requirement specifications formulated by other professional groups (e.g., nurses). Only at two wards was the system used by the physicians as intended, while at two other wards, it was used to a very limited extent, and at the last ward it was not used at all. The results thus show a correlation between the clinicians’ involvement in the decision-making during Phases 1 and 2 and positive attitudes towards the basic functionalities, along with a higher degree of use as intended [91]. With respect to the indicator: customization, the physicians and the nurses were equally positive - the physicians slightly more so. This indicator is related to the decisions taken during Phases 3 and 4, when the physicians were actively involved in the customization process. Thus, these results also stress the importance of the physicians’ involvement in decision-making with respect to attaining ownership, as well as clinical benefits as a result of these decisions.

## Discussion of methods

During Phases 1 and 2 in the evaluation of the EHR development, the authors role as evaluators meant that they were working close to the participants in the EHR working group for a longer period. This poses a risk that they were affected by group members’ attitudes - causing difficulties with keeping an objective view of the interactions between the actors. To remedy this, triangulation of methods was used together with a thorough description of all activities throughout the process to achieve the best possible transparency. Additionally, the authors were given access to all meetings during Phases 1 and 2 and to most documents related to these phases, which enabled the validation of statements from participants.

The response rate for the second survey, on which most of the results of the summative evaluation are based, varied between 35 and 77% for the physicians and between 27 and 43% for the nurses. This indicates a need for caution in generalizing the results across wards. However, they are strongly supported by the results of the focus group interviews that were carried out in all five wards at the end of Phase 4. The informants were selected by the clinicians themselves, to secure overall representation of the attitude among the respective groups of clinicians.

The experiences that formed the basis of the development of the CeHEM were gained through evaluation studies in Denmark. However, according to Berg et al., interaction between different actor groups is not a country-specific phenomenon, as it is found in eHealth development in general [95]. The CeHEM is thus capable of assisting in successful eHealth innovation, development, and implementation across borders. A barrier to the wider use of formative evaluation methods, such as the CeHEM, lies in their demands on time and money, compared to traditional summative methods. However, the gains achieved by using this method clearly compensate for this, as this paper has demonstrated.

## Conclusions

End-user involvement is an established, though rarely achieved, success factor in eHealth development. The development of the Constructive eHealth evaluation presented in this paper has been an iterative process with input from earlier research and the literature. It offers as such a full evaluation framework for the entire eHealth development lifecycle. It improves the adoption of eHealth in healthcare environments by supporting and facilitating end-user involvement during all phases. The method further offers guidance through all lifecycle phases, a modified summative evaluation, and, essentially, support for evaluation as an independent activity separated from other project activities.

The CeHEM has proven effective in evaluating EHR development in the North Denmark Region in Denmark. The evaluation made clear the consequences of failure to adhere to recommendations regarding the preconditions needed to achieve the *real* involvement of clinicians to ensure that functionalities are articulated by those who will be the primary system users, and hence to achieving joint ownership of the decisions taken during eHealth development. The recommendations are: a) Early involvement of the clinicians, b) The best possible representation of all groups of clinicians, and c) Workload reduction [50, 92]. The consequent lack ownership of the decisions taken and negative attitudes towards the clinical benefits related to the incomplete adherence. The evaluation also showed that, when the recommendations were followed and continual feedback to all actor groups was provided, the clinicians became truly involved in decision-making, which resulted in that they took ownership of decisions and gained a positive attitude to the clinical benefits related to these decisions. Finally, as a result of the evaluation, management decided to slow down the original plans for the EHR development process to ensure the best possible outcome of the process. By taking into account the feedback given by all actor groups involved, including management, problems have been avoided.

The CeHEM can be used across countries, as studies show that most interactions between user groups are not limited to one country. The method is applicable as a framework for achieving valuable information and learning during eHealth development in a broader context, thereby supporting successful eHealth development while building on one of the most well-known success factors: the true involvement of end-users in eHealth development.

## Abbreviations

AAU: Aalborg University
CeHEM: Constructive eHealth evaluation method
CPD: The competitive dialogue process
DaCHI: Danish Centre for Health Informatics, AAU
EHR: Electronic health record
ICT: Information and communication technology
